# A qualitative study on the challenges in deprescribing for elderly patients with polypharmacy

**DOI:** 10.3389/fmed.2026.1814732

**Published:** 2026-05-08

**Authors:** Weiwei Yang, Ming Li, Zhifen Wang, Qiuyang Liu, Minglian Jiang, Kangxiu Tuo, Minmin Zhang, Binyi Xia, Yong Cao, Chengli Yang

**Affiliations:** 1 Department of Pharmacy, Clinical Research Center of Integrated Traditional Chinese and Western Medicine, The Affiliated Hospital of Guizhou Medical University, Guiyang, China; 2 Department of Geriatrics, The Affiliated Hospital of Guizhou Medical University, Guiyang, China

**Keywords:** deprescribing, elderly patients, interview, polypharmacy, qualitative study

## Abstract

**Background:**

Deprescribing is a critical strategy for optimizing medication use in older adults with polypharmacy, yet its implementation in real-world clinical settings faces multifaceted challenges that require systematic understanding. This study aims to explore these barriers to inform effective service integration.

**Objectives:**

To gain an in-depth understanding of the challenges of deprescribing in elderly patients with polypharmacy and to inform the implementation of deprescribing services in healthcare institutions.

**Methods:**

Purposive sampling was utilized to carry out semi-structured in-depth interviews with key stakeholders involved in deprescribing, namely patients, physicians, pharmacists, and nurses. The data were coded, and themes were identified via the implementation of phenomenological analysis and Colaizzi’s seven-step method.

**Results:**

A total of four themes and 11 sub-themes were identified, namely clinical challenges (characterized by the complexity of managing multiple chronic conditions, the complexity of medication regimens, clinical workload, and time constraints), collaborative challenges (manifested as disruptions in interdisciplinary healthcare collaboration and a lack of trust between physicians and patients), concerns regarding medical risks (including uncertainty of treatment efficacy, safety concerns, ambiguity in responsibility and authority, decision-making inertia, and preference for the status quo), and institutional barriers (evidenced by a lack of specialized training and continuing education programs and misaligned incentive structures).

**Conclusion:**

Deprescribing for elderly patients with polypharmacy encounters numerous challenges, such as clinical intricacies, communication obstacles, apprehensions about medical risks, and institutional constraints. To facilitate the implementation of deprescribing for elderly patients with polypharmacy, a holistic reform is necessary, which involves workflow enhancement, interprofessional communication and cooperation, patient - provider interaction, and supportive systems and training initiatives. Only by clearly acknowledging the value of this work, standardizing procedures, minimizing hazards, and institutionalizing incentive mechanisms can we surmount the current barriers and allow more elderly patients to reap the benefits of safe and rational medication use and attain a peaceful and dignified aging process.

## Highlights

1. This qualitative study systematically identifies a multi-level barrier system hindering deprescribing in elderly patients with polypharmacy, encompassing clinical dilemmas, collaboration gaps, risk concerns, and institutional shortcomings.
2. A critical and often overlooked challenge is the misalignment in perceptions of “trust” among healthcare providers and patients, which fundamentally undermines team consensus and the implementation of deprescribing initiatives.
3. Overcoming these barriers requires integrated systemic reform rather than isolated improvements, including workflow optimization, interprofessional collaboration, clear responsibility frameworks, and aligned incentive mechanisms.

## 1 Introduction

The elderly population constitutes a high-risk group for chronic diseases. Approximately 75% of older adults have one or more chronic conditions, and 43% experience multimorbidity, the coexistence of multiple chronic conditions (1). Patients with multimorbidity inevitably face polypharmacy, defined as the concurrent use of five or more medications, which significantly increases the risk of inappropriate medication use, drug-drug interactions, and adverse drug reactions. These risks may lead to serious clinical consequences, including health deterioration, falls, hospitalization, and even mortality (2, 3). Deprescribing, a key component of medication reconciliation for patients on multiple medications, refers to the systematic process of reducing dosages or discontinuing medications that may cause harm or no longer provide clinical benefit (4). The primary objective is to reduce the burden and potential harms associated with medication use while preserving or improving quality of life, with its safety and effectiveness well-supported by empirical evidence (5, 6). Prescription optimization requires collaboration among multidisciplinary staff. Physicians, as the primary decision-makers, are responsible for assessing patients conditions and determining medication adjustment plans; pharmacists contribute indirectly to prescription optimization through prescription review, medication education, and medication recommendations; and nurses administer medications, assess adherence, and monitor adverse reactions. The roles and interactions of these professionals in prescription optimization directly influence the effectiveness of prescription optimization. A nationwide survey of 163 hospitals reveals that the implementation rate of medication reconciliation services in healthcare institutions is only 18.2% (7). Despite this gap, current research lacks a systematic and in-depth exploration of the barriers to deprescribing among elderly patients with polypharmacy. Compared to quantitative approaches, qualitative research enables a deeper understanding of participants’ lived experiences and subjective perspectives, yielding rich, contextually grounded insights. This study investigates the challenges encountered by physicians, pharmacists, nurses, and patients during the deprescribing process, analyzes the underlying barriers, and proposes targeted strategies and recommendations. The aim is to provide actionable guidance and a practical reference framework for healthcare institutions seeking to implement or improve deprescribing services.

## 2 Materials and methods

### 2.1 Study population

Data collection took place from July to August 2025. Using a purposive sampling methodology, this study recruited stakeholders involved in medication deprescribing-including patients, physicians, pharmacists, and nurses-from tertiary hospitals in Guizhou Province for in-depth interviews. After being referred by their attending physician, patients were recruited by the investigators through face-to-face contact; after being referred by the department head, healthcare professionals were recruited by the investigators via face-to-face or telephone contact. The sample size was determined according to the principle of data saturation, with interviews discontinued when no new themes or information emerged. This study was reviewed and approved by the Medical Ethics Committee of the Affiliated Hospital of Guizhou Medical University (Approval No. 2025177K).

Inclusion criteria: (i). Patients (P) were required to be conscious, capable of coherent and logical verbal communication, and aged 60 years or older. (ii). Doctors (D), physicians practicing in departments that commonly manage patients receiving multiple medications such as cardiology, endocrinology, neurology, and geriatrics and holding professional titles of attending physician, associate chief physician, or chief physician were included. (iii). Clinical pharmacists (C), senior clinical pharmacists, heads of the pharmacy department, or pharmacists with extensive experience in prescription review and medication education were included. (iv). Nurses (N), senior ward nurses, head nurses, and nurses working in chronic disease units, such as geriatrics and cardiology. (v). Doctors, clinical pharmacists, and nurses were required to have more than 3 years of clinical experience.

Exclusion criteria: (i). Patients with critical or severe conditions. (ii). Doctors, clinical pharmacists, and nurses who have not been previously involved in deprescribing practices in their professional experience were excluded.

### 2.2 Research methods

Prior to the formal interviews, the researchers developed preliminary interview protocols for patients, physicians, pharmacists, and nurses based on the study objectives and a comprehensive literature review. A pilot test was conducted with two participants from each group, following which the final interview protocols were refined and finalized according to the pilot test findings. Refer to Supplementary Table 2.

#### 2.2.1 Data collection methods

This study employs a descriptive qualitative research methodology, in which face-to-face, semi-structured, in-depth interviews are conducted after establishing a trust-based rapport with participants, enabling the systematic collection and thematic analysis of data. Prior to the interviews, comprehensive information regarding the purpose and significance of the study was provided to participants, and written informed consent along with audio recording authorization was obtained. The interviews were conducted in a quiet, private, and comfortable meeting room at the Affiliated Hospital of Guizhou Medical University, with each session lasting between 15 and 30 min. All interviews were audio-recorded and transcribed verbatim, and observational notes were systematically recorded to capture participants’ non-verbal behaviors, including gestures, facial expressions, and emotional responses. Audio recordings will be transcribed and systematized within 24 h after the interview, and each transcript will be assigned a unique identification code. Any omissions identified during transcription will be promptly addressed by returning to the original audio recording for clarification and supplementation.

#### 2.2.2 Data analysis methods

Apply the method of induction. The transcripts of all interviews were systematically managed in sequential order. Upon completion of audio transcription, the textual data were analyzed using the seven-step analytical framework developed by Colaizzi (8). (i). Comprehensive review of all interview transcripts; (ii). extracting meaningful statements; (iii). NVivo software was employed to support rigorous coding management; (iv). the coded perspectives were synthesized into themes; (v). A comprehensive and detailed description was provided; (vi). Common perspectives were synthesized to distill and conceptualize the core thematic essence; (vii). The analytical findings will be shared with the research participants for verification of content authenticity. The specific procedure is as follows: After identifying the themes, the researcher selected a total of five participants—two patients, one physician, one pharmacist, and one nurse—from the four participant groups to conduct member validation. Through face-to-face discussions, the researcher presented the identified themes to them in plain language and asked whether the findings accurately reflected their actual experiences. The feedback was discussed by the research team and incorporated into the final analysis results.

#### 2.2.3 Quality control method

The interviews were conducted independently by research personnel who had completed preliminary training in qualitative research methodology prior to data collection and demonstrated competence in effective communication with both patients and healthcare providers. The study employed a semi-structured in-depth interview approach, conducted in a private setting to ensure a quiet and comfortable environment free from external interruptions while maintaining participants’ confidentiality. Prior to formal data collection, pilot interviews were conducted to refine and finalize the interview protocol. During the interview process, participants were encouraged to fully express their authentic thoughts and feelings, while leading or suggestive questions were avoided. Exploratory questions were used strategically to elicit comprehensive and nuanced data.

## 3 Results

A total of five patients, six physicians, four pharmacists, and four nurses participated in the study. The general characteristics of the participants are presented in Table 1. Upon reviewing and analyzing the interview data, the researchers identified and synthesized four primary themes and eleven sub-themes.

**TABLE 1:** Baseline characteristics of survey respondent.

No.	Category	Gender	Age	Profession	Educational level	Title	Years of service	Duration (minutes)
P1	Patient	Male	62	Teacher	Undergraduate	Senior teacher	Retired	28
P2	Patient	Female	65	Accountant	Secondary	Accountant	Retired	26
P3	Patient	Female	70	Worker	Junior	/	Retired	24
P4	Patient	Male	73	Farmer	Primary	/	/	29
P5	Patient	Female	81	Farmer	Primary	/	/	33
D1	Doctor	Female	34	Cardiovascular medicine	Master	Attending doctor	8	21
D2	Doctor	Female	43	Respiratory medicine	Master	Associate doctor	19	27
D3	Doctor	Male	33	Nephrology	Master	Resident doctor	7	22
D4	Doctor	Female	37	Gastroenterology	Master	attending doctor	11	28
D5	Doctor	Male	48	Neurology	Doctor	Senior doctor	23	21
D6	Doctor	Female	45	Endocrinology	Master	Associate doctor	20	29
C1	Clinical Pharmacist	Female	32	Pharmacy	Undergraduate	Pharmacist	8	25
C2	Clinical pharmacist	Male	37	Clinical pharmacy	Doctor	Associate chief pharmacist	6	19
C3	Clinical pharmacist	Female	56	Clinical pharmacy	Undergraduate	Associate chief pharmacist	30	22
C4	Clinical pharmacist	Female	48	Clinical pharmacy	Master	Chief pharmacist	24	16
N1	Nurse	Female	28	Cardiovascular medicine	Junior	Nurse	8	21
N2	Nurse	Female	35	Endocrinology	Undergraduate	Supervisor nurse	11	18
N3	Nurse	Female	36	Neurology	Undergraduate	Supervisor nurse	12	21
N4	Nurse	Female	34	Gastroenterology	Master	Supervisor nurse	7	27

### 3.1 Theme 1: clinical dilemmas

#### 3.1.1 The complexity of managing multimorbidity

Multimorbidity in elderly patients often necessitates polypharmacy, posing significant clinical challenges for healthcare providers in implementing appropriate prescribing and deprescribing strategies—a reflection of the inherent complexity involved in managing multiple coexisting chronic conditions. “The patient was admitted to the hospital due to inadequate disease control. For cases with a clearly identifiable cause, precise treatment can typically be achieved. However, in cases where the underlying etiology remains unclear, clinical decision-making may prioritize the addition of medications to rapidly alleviate current symptoms over dose reduction (D3).” “Patients with multiple comorbidities often present diagnostic challenges due to the difficulty in differentiating latent adverse drug reactions from symptoms of underlying medical conditions. For example, an elderly patient receiving polypharmacy for hypertension and diabetes reported severe fatigue and dizziness. Given the coexistence of coronary artery disease and cerebrovascular disease, it was initially unclear whether these symptoms represented disease progression. Such as myocardial ischemia or cerebral hypoperfusion or were attributable to adverse drug effects, including hypotension or hypoglycemia resulting from drug interactions or inappropriate dosing (D4).”

#### 3.1.2 The complexities of polypharmacy management

The complexity of deprescribing extends beyond merely managing the polypharmacy embedded in therapeutic regimens; it is further compounded by intricate procedural dynamics, which impose substantial demands on healthcare professionals’ clinical judgment as well as patients’ health literacy and adherence capacity. “The patient is prescribed multiple medications, requiring a comprehensive evaluation based on clinical indications, dosage regimens, treatment duration, and drug-related risks to identify those amenable to regimen simplification, a task that presents considerable clinical complexity (D1).” “A clinical case involved a patient prescribed an oral regimen consisting of 22 distinct medications, including three antidiabetic agents, including metformin, acarbose, and empagliflozin, despite persistent suboptimal glycemic control. Given the complexity of insulin pen administration, which the patient reported difficulty in learning and consistently performing, a transition to insulin therapy was recommended. However, the patient ultimately elected to continue with the existing oral medication regimen (D3).” “Clinical prescription optimization can be effectively applied to outpatient prescriptions and discharge medications. However, over-the-counter health supplements and self-obtained proprietary Chinese patent medicines may be inadvertently overlooked, as these agents are often excluded from standardized medication reconciliation processes (C2).”

#### 3.1.3 Clinical workload and time pressure

In time-constrained clinical settings, there is a lack of adequately allocated temporal and operational resources to support comprehensive prescription optimization. “Amid the time constraints of outpatient consultations, the complexity of diverse medical conditions and intricate medication regimens present significant challenges to the systematic organization of clinical information and the development of streamlined prescription plans (D1).” “I have 30 patients to see in the morning outpatient clinic. It’s already very busy to take care of all these 30 patients. I simply don’t have the time or energy to simplify the prescriptions (D2).” “The number of patients each of our nurses is responsible for is already at an overloaded level, making it difficult to fully monitor their drug reactions. Many times, we just ask if they feel any discomfort and then move on (N1).” “Working at the service window with long queues of patients, there is often no time to identify duplicate medications or potential drug interactions from a multitude of bottles. What I can ensure is the strict implementation of verification protocols to guarantee that “the right medication is administered to the right patient (C1).”

### 3.2 Theme 2: the collaborative challenges

#### 3.2.1 Process discontinuities in multidisciplinary healthcare collaboration

The implementation of deprescribing faces practical challenges arising from disruptions in interprofessional collaboration processes, primarily manifested as omissions and delays in the transmission of critical information during cross-disciplinary patient handovers. “As licensed pharmacists without prescriptive authority, we can only provide medication optimization recommendations to patients. However, we cannot verify whether such advisories are effectively communicated to or accurately relayed by patients when they consult physicians for prescription adjustments (C2).” “The pharmacist team provides highly professional medication counseling to patients. However, when patients arrive at my consultation room, the information they relay is often vague and imprecise, for example: “The pharmacist mentioned that the white pill might not be suitable” or “Someone said taking this medication excessively could harm the kidneys.” In such cases, it is typically unclear which pharmacist provided the advice, what specific concerns were raised, or on what clinical evidence or rationale the recommendations were based (D2).” “I typically inform the physician immediately of a patient’s medication-related reactions. However, regarding pharmacist recommendations, due to the inconsistent presence of pharmacists across departments and the lack of a standardized schedule for pharmacy rounds, information is often relayed indirectly through messages left for the attending physician (N1).”

#### 3.2.2 The erosion of trust in the doctor-patient relationship

In the collaborative effort to streamline prescribing practices, pharmacists and nurses perceive that patients place trust primarily in their attending physicians, whereas physicians observe that patients tend to place greater confidence in senior and renowned specialists. This divergence in perceptions regarding the attribution of trust creates a climate of caution among all parties, who remain hesitant to advance streamlining initiatives in the absence of strong authoritative endorsement. “Most patients tend to adhere to physicians’ recommendations, whereas deprescribing proposals put forward by pharmacists are often not readily accepted. This disparity stems from the prevailing perception among patients that physicians possess greater professional authority and clinical credibility compared to pharmacists (C3).” “Patients are generally more inclined to follow physicians’ recommendations and may perceive nursing staff as having less authority in clinical decision-making. When implementing a physician’s order to discontinue medication, healthcare providers occasionally encounter situations in which patients secretly retain their medication, believing that the omission was initiated by the nurse rather than being part of a formal medical directive from the physician (N1).” “Most patients place greater trust in senior physicians. When prescriptions are issued in outpatient settings, many patients explicitly emphasize that their medication regimens were established by a specific chief physician (D2).” “If a physician recommends discontinuing a medication, I would seriously consider doing so. When collecting prescriptions at the pharmacy, pharmacists typically provide guidance on medication use and associated precautions. Regarding any recommendations they offer, I would first consult my physician rather than adopt them directly (P1).” “I only follow the advice of my attending doctor regarding whether to simplify my prescriptions or not. My heart surgery was performed at Fuwai Hospital in Beijing, which is the top hospital for heart diseases in China. All the medications I take are prescribed by the doctors there, and I won’t stop taking them casually (P3).”

### 3.3 Theme 3: medical risk considerations

#### 3.3.1 The uncertainty regarding the therapeutic effect

The medical risk considerations associated with the process of deprescribing arise from a cautious assessment of the uncertainty surrounding treatment effects; the recurrence of disease symptoms represents the primary clinical concern, which aligns closely with patient concerns. “Following medication discontinuation in the patient, the primary concern is the potential recurrence of the disease. Should recurrence occur, the patient may question the clinical decision made by the physician at that time (D1).” “If the treatment outcome fails to meet the patient’s expectations following deprescribing, the risk of the patient experiencing negative emotional responses will increase significantly; such circumstances may constitute a potential risk for medical disputes (D2).” “I also believe that I am currently taking an excessive number of medications. I am concerned that discontinuing these medications may negatively impact the control of my blood pressure and blood glucose levels (P1).”

#### 3.3.2 Safety considerations

Prescription streamlining is considered a potential health risk, with perceived risks primarily centered on concerns regarding loss of disease control and uncertainties surrounding the tolerability of newly introduced medications. “For elderly patients who have been on long-term sedative-hypnotic agents (e.g., estazolam) or anti-anxiety medications (e.g., paroxetine), abrupt discontinuation or rapid dose reduction may lead to severe withdrawal syndromes, such as heightened anxiety, insomnia, and palpitations or a rebound of the underlying condition, potentially more severe than the pre-treatment state. Therefore, deprescribing involving these medications will not be pursued (D3).” “I dare not stop taking any of my antihypertensive drugs. Back then, the doctor said my blood pressure was alarmingly high. It took me so many years to stabilize it after taking the medicine. What if I stop taking them and my blood pressure shoots up all of a sudden? I might have a stroke. How could I deal with that? (P3).” “The doctor suggested that I replace these four or five kinds of medicine with a compound preparation. He said the effect would be the same and it could also reduce the burden. But I’m already used to these medicines I’m taking now and my body has no adverse reactions. If I switch to a new medicine, who knows if I’ll be allergic or have some other side effects? I don’t want to change (P5).”

#### 3.3.3 Boundary of rights and responsibilities

The implementation of deprescribing faces professional risk concerns arising from ill-defined authority and responsibility boundaries, primarily reflected in healthcare providers’ apprehensions about exceeding their scope of practice and the uncertainty surrounding accountability in interdisciplinary collaboration. “I have consistently worked in a specialized clinical department, where we have extensively discussed the onset, progression, and ultimate treatment outcomes of common conditions within our specialty. I am highly familiar with the appropriateness of medication use and the accuracy of dosing regimens, making it entirely appropriate for me to undertake deprescribing within this domain. However, regarding other specialized departments, my knowledge is limited, and therefore I refrain from offering opinions beyond my expertise (D5).” “When patients express doubts regarding a physician’s decision to discontinue medication, it is typically the physician who provides clarification. Our professional responsibility centers on monitoring and documenting patient responses. Therefore, it is more appropriate for the prescribing clinician to explain the rationale for medication cessation (N2).” “Deprescribing entails adjusting medication regimens based on an individual patient’s clinical condition. As pharmacists, we typically lack the comprehensive understanding of patients’ overall health status that physicians possess; therefore, we do not routinely initiate recommendations for deprescribing directly to patients (C3).”

#### 3.3.4 Decision inertia and *status quo* bias

Healthcare providers and patients frequently view the continuation of the current prescription regimen as the safer “default choice.” This risk-averse cognitive bias diminishes their willingness to initiate deprescribing, thereby contributing to decision-making inertia that perpetuates the *status quo*. “In my outpatient clinic, approximately 5% of patients proactively request a simplified prescription. For the remaining patients, I typically do not initiate a medication review unless there is clear evidence of duplicate therapy or the patient reports adverse reactions, in which case I will advise discontinuation of the affected medication (D1).” “Some patients consistently follow the original prescription during each follow-up visit. This prescription was established by the former department head and has remained in use for several years. In the absence of reported significant discomfort, I generally continue the existing medication regimen (D2).” “I recognize that there is potential for optimizing the patient’s medication regimen. However, performing a comprehensive assessment, reviewing relevant literature, and engaging in further communication with the physician would require substantial time and effort. In the absence of an active consultation request from the treating clinician, such cases are typically deferred (C4).”

### 3.4 Theme 4: lack of management

#### 3.4.1 The absence of implementation standards and continuing education system

As a clinical operational procedure, deprescribing has not yet established mandatory implementation standards at the national level, and healthcare institutions likewise lack a dedicated continuing education system in this area. “We can identify inappropriate medications in prescriptions; however, due to the lack of systematic training in medication streamlining, we are unable to develop comprehensive, reliable, and persuasive alternative treatment plans for both physicians and patients (C3).” “Due to the lack of specialized training in deprescribing, I am uncertain about appropriate post-deprescribing interventions and key nursing priorities in patient care (N1).” “Upon joining the geriatrics department, I was first introduced to the concept of deprescribing. Pharmacotherapy for older adults should prioritize medication reduction rather than the continuous addition of drugs. During my previous clinical experience in other medical specialties, I had not received systematic training in deprescribing principles (D5).”

#### 3.4.2 Misalignment of incentive mechanisms

Prescription streamlining is a highly professional and time-intensive clinical intervention. Within the current fee-for-service healthcare payment system and performance evaluation frameworks emphasizing service volume, the value of this work is frequently underrecognized and seldom independently measured or adequately reflected. Healthcare professionals invest considerable time in clinical duties yet often do not receive commensurate performance-based compensation, which is likely to undermine their motivation. Under the prevailing performance accounting model, physician remuneration is frequently tied to metrics such as patient volume and surgical caseload. “In contrast, the effort required to communicate with patients and their families, particularly when justifying the discontinuation of long-term medications is significantly greater than that involved in routine prescription renewal, yet it remains underweighted in incentive structures (D2).” “Clinical pharmacists participate in medication adjustments through consultative services; however, the absence of a performance-based incentive mechanism limits their active engagement. As a result, relatively few clinical pharmacists take the initiative to participate (C2).” “Following the discontinuation or modification of medications, it is necessary to increase the frequency of monitoring patients’ vital signs and subjective symptoms, with close attention to potential withdrawal reactions or disease relapses. However, when reporting workload, these additional clinical efforts cannot be quantified and are therefore not reflected in performance evaluations, which inevitably affects professional motivation (N3).”

## 4 Discussion

Deprescribing constitutes an active and patient-centered approach to optimizing medication regimens through collaborative decision-making between clinicians and patients (9). Although a substantial body of existing research has demonstrated the safety and feasibility of deprescribing, its implementation rate in China remains relatively low (10). This study focuses on the difficulties and challenges encountered in the process of deprescribing, examining key factors that constrain the implementation of deprescribing practices from the perspectives of physicians, pharmacists, nurses, and patients.

### 4.1 Healthcare professionals’ efforts in deprescribing are influenced by a range of multifaceted factors, including constraints in clinical practice, limitations in time and resource allocation, variations in professional expertise, and institutional protocol barriers

The complexity of managing comorbidities and polypharmacy in geriatric patients significantly increases the challenges associated with medication regimen optimization. Deprescribing is widely recognized as a time-intensive process. A study conducted by Dr. Zhen Jiancun’s team found that the duration of a single medication review session ranges from 14.2 to 27.4 min (7). Globally, the average consultation duration for primary care physicians varies significantly, ranging from 48 s to 22.5 min (11–13). In time-constrained clinical settings, healthcare professionals face significant challenges in allocating sufficient time and resources to implement medication deprescribing effectively. The current supply–demand imbalance aligns with established findings in the international literature. Notably, Brunner et al.’s systematic review identifies lack of time as a primary barrier to prescription simplification (14). Consistent with the findings of Moth et al. indicated that physicians’ subjective perceptions of medications and their beliefs regarding the potential negative consequences of discontinuation pose significant barriers to prescription streamlining (15). Building on this, the present study further reveals the influence of professional background; constrained by their professional background, physicians often prioritize medication safety as their primary consideration when streamlining the use of drugs outside their specialty. Driven by cautious assessments of therapeutic uncertainty and potential risks, they are more likely to maintain existing prescriptions, thereby actively avoiding cross-specialty deprescribing. Previous studies have demonstrated that pharmacists’ expertise in pharmacology, drug interactions, and prescription review is essential in the context of deprescribing (16). However, in contrast to the emphasis in the literature that pharmacists “should play a more active role,” This study reveals that due to clinical information asymmetries and constraints arising from the lack of statutory prescriptive authority, pharmacists primarily participate in deprescribing through consultative recommendations rather than by proactively initiating deprescribing protocols. This finding reveals how institutional factors constrain the role of pharmacists Mardani et al. found that nurses contribute to medication management hrotugh their involvement in the medication verification process (17). This study further confirms this viewpoint. Nurses typically conceptualize their professional role primarily in terms of task execution and monitoring, rather than clinical interpretation and decision-making, and rarely initiate deprescribing activities proactively. This finding suggests that the potential value of nurses in prescription simplification has not yet been fully realized. Hickman et al. further observed that the potential contribution of nurses to prescription simplification remains underutilized (18). The absence of a systematic deprescribing training framework and corresponding incentive mechanisms within healthcare institutions significantly reduces the engagement of medical personnel in deprescribing initiatives. Zhao et al.’s study further indicates that successful implementation of prescription simplification guidelines is hindered by multiple interrelated barriers, notably inadequate training and limited resources (19).

### 4.2 Patient acceptance of deprescribing is primarily influenced by uncertainties about disease control and concerns regarding adverse effects associated with medication changes

Reeve et al. have demonstrated that patients frequently report concerns about taking multiple medications, and over 90% of patients are theoretically willing to discontinue medications upon physician recommendation (20). Consistent with this high level of willingness, all five interviewed patients indicated that polypharmacy posed significant challenges to their daily medication management. Under medical guidance, they expressed willingness to discontinue unnecessary medications. Although patients theoretically are willing to accept the simplification. Clinical data indicate that patients continue to demonstrate resistance to prescription streamlining in practical implementation (21, 22). Anderson et al. identified fear of unknown clinical variability and potential adverse outcomes as barriers to deprescribing potentially inappropriate medications in patients (23). Consistent with these findings, this study found that patients’ primary concerns centered on the potential disruption of current disease stability and apprehensions about unknown side effects associated with the new medication regimen. Van Leeuwen et al.’s systematic review further confirms that patients concerns regarding disease recurrence or clinical deterioration constitute a primary barrier to treatment discontinuation decisions (24). This is highly similar to the concerns of clinical doctors regarding risks, suggesting that there is convergence in risk perception between doctors and patients.

### 4.3 The clinical implementation of deprescribing faces two major obstacles: discontinuities in interprofessional collaboration and a lack of trust in the physician–patient relationship

Deprescribing requires close collaboration among clinicians, pharmacists, and other healthcare professionals, integrating clinical evidence, professional expertise, and patient-specific factors into a comprehensive assessment. Reidt et al. conducted a study on an interprofessional collaboration model for medication management, which highlights the critical importance of collaborative practices among physicians, pharmacists, and nurses (25). Collaborative and Co-Ordinated Action for Medication Safety (COMS). indicate that effective communication between healthcare professionals and patients is a critical component of medication safety (26). Based on the aforementioned basic principles of cross-disciplinary collaboration, this study reveals that pharmacists’ prescription review recommendations in the context of deprescribing are vulnerable to information distortion and sourcing from unverified channels when transmitted through patients, thereby impairing physicians’ ability to track and trust these professional judgments. Concurrently, nurses’ bedside observations are subject to delayed and indirect communication with physicians and pharmacists, which hinders the establishment of an efficient closed-loop system and results in missed opportunities for critical interventions. These findings specifically reveal the communication obstacles that previous research has highlighted as potentially arising in cross-professional collaboration in practice. Evidence from previous studies indicates that patients’ level of trust in their physicians significantly influences their willingness to accept and adhere to deprescribing recommendations (27). Ie et al.’s study further indicates that patients are more likely to accept prescription simplification when guided by clinicians they trust (28). This study reveals that pharmacists and nurses perceive physicians as the primary recipients of patient trust, whereas physicians recognize patients’ tendency to place greater confidence in higher-level medical authorities. This asymmetry in perceptions of the trust hierarchy fundamentally undermines the foundation for achieving consensus within healthcare teams during the implementation of deprescribing initiatives. This asymmetrical perception of the trust structure further undermines the foundation on which the medical team can reach consensus during the process of implementing prescription simplification. This aspect has not been adequately addressed in previous literature.

## 5 Conclusion

This study employed a phenomenological approach to systematically explore the barriers encountered in the process of prescription simplification for elderly patients on multiple medications through in-depth interviews with four categories of stakeholders: physicians, pharmacists, nurses, and patients. It identified complex influencing factors across multiple dimensions, including clinical dilemmas, interprofessional collaboration, trust relationships, and management systems. The study sample was drawn exclusively from a single Grade A tertiary hospital in Guizhou Province, comprising 19 participants. As a phenomenological study, we sought a deep understanding of lived experiences within a specific context rather than generalizability in a statistical sense; therefore, the transferability of the findings to other contexts should be evaluated with caution. The research team consisted of clinical pharmacists and qualitative methodology experts. Although diverse backgrounds facilitate a multi-perspective understanding, they may also introduce preconceptions. To address this, we employed methods such as team discussions and independent coding during the analysis process to ensure balance. Due to clinical work schedules and the participants’ physical conditions, each participant underwent only one interview (15–30 min); a single interview may not fully capture the dynamic changes in participants’a experiences. Furthermore, this study focused on barriers at the clinical practice level and did not delve deeply into the impact of hospital management systems (such as incentive mechanisms for prescription simplification and institutionalized processes for interprofessional collaboration) on the implementation of prescription simplification. Future research could adopt a longitudinal design to track the interactive processes of prescription simplification decisions through multiple interviews; it could also be expanded to healthcare institutions at different levels and in different regions to test the generalizability of the findings. It is recommended to further explore, from the perspective of management systems, how policy design and institutional optimization can address the practical barriers identified in this study. Despite these limitations, through a multi-perspective analysis of four categories of stakeholders, this study provides an important empirical foundation for future research on multidisciplinary collaborative interventions for prescription simplification.

## Data Availability

The raw data supporting the conclusions of this article will be made available by the authors, without undue reservation.
