# Supplementary material for: A qualitative study on the challenges in deprescribing for elderly patients with polypharmacy
Source: Front Med (Lausanne). 2026 May 8;13:1814732. doi: 10.3389/fmed.2026.1814732 (PMC13193912; doi:10.3389/fmed.2026.1814732)
Supplement: Supplementary file 1 [file Data_Sheet_1.pdf]

Supplementary Materials

Table 2. Interview Protocol Outline

Interviewee	Outline
Doctor	1. In your clinical practice, what proportion of patients do you estimate are affected by polypharmacy, that is, the concurrent use of five or more medications—and how do you assess this phenomenon?<br>2. Is "deprescribing" a clinical strategy that you actively consider in your daily practice? Under what circumstances would you consider initiating deprescribing for a patient?<br>3. What is your primary clinical concern when deciding whether to discontinue a particular medication?<br>4. If adverse consequences may arise from drug discontinuation, could the potential risk of medical disputes influence your clinical decision-making? How do you weigh the benefits against the risks?<br>5. How do you manage the coordination challenges between "specialized prescribing" and "holistic patient-centered medication management"?<br>6. How do you evaluate the phenomenon of "prescription inertia"—defined as the habitual continuation of the original prescription without reassessment of the patient's condition? To what extent does it represent a challenge in clinical practice?<br>7. What are the most common concerns expressed by patients when discontinuing a long-term medication regimen?<br>8. In your assessment, does the current healthcare system facilitate or hinder the implementation of deprescribing?<br>9. What specific incentives do you believe hospitals or medical insurance policies should provide to encourage physicians to dedicate more time to medication review and deprescribing efforts?<br>10. Are there any other important aspects of this issue that have not been addressed but should be considered?
Clinical Pharmacist	1. In your opinion, what is the unique and irreplaceable role of pharmacists in multidisciplinary team collaboration for "deprescribing"?<br>2. How are you currently involved in prescription streamlining? Which model of participation do you consider to be the most effective?<br>3. When you propose deprescribing to a physician, what are the most common reasons for your suggestions being rejected or overlooked?<br>4. What are the primary technical challenges you encounter when determining which medications should be discontinued?<br>5. What are the common forms of patient resistance encountered during deprescribing? Which inherent patient beliefs or concerns do you find most challenging to address?

	6. When providing deprescribing recommendations, do you frequently encounter challenges in ensuring their safety due to incomplete patient information?
	7. Does the current performance appraisal system adequately recognize your efforts in deprescribing? Is there a perceived risk of greater accountability or adverse consequences with increased intervention, leading to the perception that non-intervention may be safer?
	8. When proactively adjusting long-standing prescriptions, do you experience psychological stress or professional hesitation that you must overcome?
	9. How would you describe the level of frustration you experience when a deprescribing plan, to which you have devoted significant effort, is ultimately rejected or not implemented? What strategies do you typically use to manage your emotional response and maintain professional effectiveness?
	10. If you were asked to rank the challenges listed above according to their difficulty to address, which one or two would you consider the most significant barriers to overcome?
	11. To address the challenges described above, what single improvement measure do you consider most critical to implement?
Nurse	1. What are the main logistical challenges faced by patients under your care who take five or more medications?
	2. Have patients who have experienced the challenges described above ever requested deprescribing from you?
	3. When implementing a "treatment discontinuation" order, what are the most common patient reactions and questions? How do you typically explain the clinical rationale and engage in communication with them?
	4. If the decision to discontinue medication is made by a particular subspecialty while the patient is simultaneously receiving care from another, have you encountered situations in which clinicians from the other specialty question this decision? How do you typically manage the coordination challenges associated with differing clinical opinions across specialties?
	5. Have you ever observed patients exhibiting unusual symptoms after medication discontinuation, but found it difficult to determine whether these were related to the withdrawal? How do you typically communicate such uncertain clinical observations to physicians or pharmacists?
	6. When clinical indicators suggest that a patient may benefit from deprescribing, what is your typical approach to communicating recommendations to physicians? To what extent are these recommendations valued and acted upon in clinical practice?
	7. From your perspective, are there information gaps or communication delays in the deprescribing process among physicians, pharmacists, and nurses? Which step in the process requires the most improvement?
	8. The need for intensive monitoring and communication with patients undergoing medication discontinuation undoubtedly increases clinical workload. How do you typically manage this challenge given the current

	demands of clinical nursing practice?<br>9. Do you believe the ward lacks a standardized nursing protocol for "post-discontinuation monitoring"? If so, has this gap led to practical challenges in your clinical practice?<br>10. Have you received formal training on the principles and precautions associated with deprescribing? If so, do you consider such training to be necessary?<br>11. Drawing on your frontline professional experience, what do you consider the most critical barrier to be for the successful implementation of deprescribing?<br>12. What recommendations do you have for enhancing nurses' role in the deprescribing process?
Patient	1. Could you elaborate on what you consider to be the most challenging aspect of managing multiple medications in daily clinical practice?<br>2. Have these medications caused any significant disruptions to your daily life, including physical discomfort or financial strain?<br>3. What was your primary psychological response when a physician or pharmacist initially suggested that you consider reducing or discontinuing certain medications?<br>4. What are your primary considerations or concerns that you would like to address before deciding whether to accept the deprescribing recommendation?<br>5. Based on what criteria do you assess whether to trust deprescribing recommendations from physicians or pharmacists? What specific information or actions could strengthen your confidence in and willingness to accept such recommendations?<br>6. In the event of conflicting medical opinions, such as one physician recommending discontinuation of a medication while another advises continuing treatment, do you experience uncertainty or hesitation? How do you typically approach decision-making in such situations?<br>7. Could the abrupt discontinuation of long-term medication potentially lead to psychological discomfort or a loss of sense of security?<br>8. Following discontinuation of the medication, do you pay particular attention to physical changes? Have you ever attributed minor discomforts to the process of stopping the medication?<br>9. During the process of medication tapering or discontinuation, what types of support and assistance do you most hope to receive from physicians or pharmacists?<br>10. What would you consider to be an ideal medication tapering process?<br>11. What roles do you expect physicians, pharmacists, and nurses to play during the medication tapering process?<br>12. Are there any other aspects of your medication use that you consider important but were not covered in our discussion so far?
